# Virus infection facilitates the development of severe pneumonia in transplant patients with hematologic malignancies

**DOI:** 10.18632/oncotarget.10182

**Published:** 2016-06-20

**Authors:** Caifeng Yue, ZhiJie Kang, Kexin Ai, Duorong Xu, Jim Wu, Yujia Pan, JinSong Yan, Min Liu, Quentin Liu

**Affiliations:** ^1^ Department of Hematology, The Third Affiliated Hospital, Sun Yat-sen University, Guangzhou, China; Department of Laboratory Medicine, The First Affiliated Hospital of Sun Yat-sen University, Guangzhou, China; Institute of Cancer Stem Cell, Dalian Medical University, Dalian, China; ^2^ Department of Hematology, The Second Affiliated Hospital, Dalian Medical University, Dalian, China; ^3^ Department of Hematology, The First Affiliated Hospital, Sun Yat-sen University, Guangzhou, China; ^4^ Roche Pharmaceutical Research and Early Development, Shanghai, China

**Keywords:** cytomegalovirus (CMV), respiratory syncytial virus (RSV), co-infection, severe pneumonia, hematologic malignancies

## Abstract

Allogeneic hematopoietic stem cell transplantation (HSCT) is an effective therapy for patients with hematologic malignancies. Severe pneumonia is associated with high mortality rate in HSCT recipients. Viral co-infection indicates a poor prognosis of HSCT recipients. In this study, a total of 68 allogeneic HSCT recipients were included. Cytomegalovirus (CMV) and Respiratory syncytial virus (RSV) infection was assessed by testing peripheral blood and oropharynx swabs, respectively, collected in the first 180 days after transplantation. We analysed the correlation of CMV and RSV co-infection with severe pneumonia and mortality. The incidence of CMV and RSV co-infection was 26.5% (18/68). Severe pneumonia was diagnosed in 61% (11/18) cases with co-infection compared to only 10% (5/50) cases with mono-infection or no infection. The analysis of potential risk factors for severe pneumonia showed that CMV and RSV co-infection was significantly associated with severe pneumonia (*p* < 0.001). The 5 patients who died of severe pneumonia were all co-infected with CMV and RSV. In conclusion, CMV and RSV co-infection appears to be an important factor and facilitates the development of severe pneumonia in allogeneic HSCT patients with hematologic malignancies.

## INTRODUCTION

Hematopoietic stem cell transplantation (HSCT) is primarily used in the treatment of hematological malignancies [1]. Despite the improved outcomes of HSCT, patients remain at high risk for potentially fatal infections due to immunosuppression after engraftment. Pneumonia is a frequent infectious complication, and development of severe pneumonia is a common cause of mortality after allogeneic HSCT [2, 3]. Although many microorganisms are associated with pneumonia, viruses are recognised as the predominant pathogens leading to pneumonia after allogeneic HSCT [4, 5].

Cytomegalovirus (CMV) is a ubiquitous herpes virus and the prevalence of CMV infection (anti-CMV IgG positive) in Chinese blood donors is 98.5% [6]. CMV seropositivity and/or documented reactivation have been associated with morbidity and mortality after HSCT [7]. The majority of patients are at risk for CMV reactivation after HSCT, which results in multi-organ inflammation and an increased rate of graft-versus-host disease (GVHD) [8, 9]. Additionally, recent studies have identified other viruses, such as respiratory syncytial virus (RSV), Parainfluenza virus (PIV), Influenza virus, and human Metapneumovirus (MPV), as important factors contributing to pneumonia in HSCT recipients [10]. Respiratory syncytial virus (RSV) is a common cause of seasonal respiratory viral infections in patients who have undergone HSCT [11]. In susceptible young children and immunosuppressed elderly individuals, RSV can exploit host immunity and cause a strong inflammatory response that leads to lung damage and viral dissemination [12–14]. In immunocompromised hosts, RSV infection usually manifests as an upper respiratory illness (URI) that frequently progresses to a lower respiratory illness [15, 16].

Viral co-infections have been noted to increase risks of mortality in HSCT recipients [17, 18]. Previous studies have investigated the risk factors for either RSV or CMV infection [7, 19–22], but none of them examined the risk factors and outcomes of CMV and RSV co-infection in HSCT patients. This prospective study was conducted to investigate the presence of two viruses associated with the development of severe pneumonia among 68 allogeneic HSCT patients. The results indicate that CMV and RSV co-infection facilitated the development of severe pneumonia in allogeneic hematopoietic stem cell transplant patients with underlying hematologic malignancies.

## RESULTS

### Patients' preliminary characteristics

The patients' preliminary characteristics are shown in Table 1. The median patient age was 29.4 years, and the patients were predominantly male (43/68; 63.2%). In the majority of cases, the underlying diseases were acute myelocytic leukaemia (37/68; 51.5%) and acute lymphocytic leukaemia (25/68; 36.8%). The most common HLA (human leukocyte antigen) donor type was matched related donor (45/68; 66.2%). Patient stem cells were most commonly derived from the peripheral blood (43/68; 63.2%). Thirteen patients (13/68; 19.1%) were diagnosed with Grade 3-4 Acute GVHD or Chronic GVHD, 55 patients (55/68; 80.9%) were diagnosed with Grade 0-2 GVHD.

**Table 1:** Characteristics of allogeneic HSCT recipients

Characteristic	Total
	(N=68)
	N (%)
**Age years : mean (s.d.)**	29.4 (10.9)
**Gender**	
Female	25 (36.8)
Male	43 (63.2)
**Underlying disease**	
Acute myelocytic leukemia	37 (51.5)
Acute lymphoblastic leukemia	25 (36.8)
Chronic myelocytic leukemia	2 (4.4)
Lymphoma	1 (2.9)
Mixed lineage leukemia	3 (4.4)
**Donor type**	
Matched related	45 (66.2)
Matched unrelated	13 (19.1)
Mismatched related	5 (7.4)
Mismatched unrelated	5 (7.4)
**Stem cell source**	
Peripheral blood	43 (63.2)
Bone marrow	8 (11.8)
Bone marrow and peripheral blood	17 (25.0)
**GVHD**	
Acute GVHD Grade 0-2	55 (80.9)
Acute GVHD Grade 3-4 or Chronic GVHD	13 (19.1)

### Incidence of CMV and RSV mono-infection and co-infection

The CMV serology status of the recipients and donors was detected before HSCT. Positive for CMV-IgG and negative for CMV-IgM were confirmed for all of recipients and donors before HSCT. A total of 437 peripheral blood samples were collected and tested for CMV, a mean of 6.4 (4-14) samples per patient. A total of 68 patients had 131 episodes of CMV reactivation during the study period, with a mean of 1.9 (0-8) episodes per patient. A total of 437 oropharynx swabs were collected and tested for RSV, with a mean of 6.4 (3–15) per patient. The 68 patients had 88 episodes of RSV infection during the study period (mean 1.3 episodes, ranging from 0-5 episodes per patient).

The incidence of CMV and RSV co-infection was 26.5% (18/68) in patients with allogeneic HSCT. The 18 co-infected patients had only one episode of CMV and RSV co-infection during the course of the study. The incidences of either CMV or RSV mono-infection were 48.5% (33/68) in allogeneic HSCT patients. CMV mono-infection was identified in 29.4% (20/68) of allogeneic HSCT patients. RSV mono-infection was identified in 39.7% (27/68) of allogeneic HSCT patients. Neither CMV nor RSV infection was tested in the rest of the 17 allogeneic HSCT patients. Patients who had co-infection, mono-infection, and no infection shared common characteristics including age, gender, underlying diseases, donor type, and stem cell source (*p* = 0.095, *p* = 0.258, *p* = 0.95, *p* = 0.283, *p* = 0.496). However, the three groups differed significantly in the presence of acute GVHD grade 3-4 or chronic GVHD (*p* < 0.001, Table 2 ).

**Table 2:** Characteristics of allogeneic HSCT patients with and without CMV/RSV infection

Characteristic	Co-infection	Mono-infection	No infection	*p^a^*
	(N=18)	(n=33)	(n=17)	
	N (%)	N (%)	N (%)	
**Age** · **years : mean (s.d.)**	33.2 (10.2)	26.9 (11.3)	30.3 (10.9)	0.095*^b^*
**Gender**				0.258
Female	5 (27.8)	11 (33.3)	9 (52.9)	
Male	13 (72.2)	22 (66.7)	8 (47.1)	
**Underlying disease**				0.95
Acute myelocytic leukemia	10 (55.6)	16 (48.5)	11 (64.7)	
Acute lymphoblastic leukemia	6 (33.3)	14 (42.4)	5 (29.4)	
Chronic myelocytic leukemia	1 (5.6)	1 (3.0)	0 (0.0)	
Lymphoma	0 (0.0)	1 (3.0)	0 (0.0)	
Mixed lineage leukemia	1 (5.6)	1 (3.0)	1 (5.9)	
**Donor type**				0.283
Matched related	15 (83.3)	18 (54.5)	12 (70.6)	
Matched unrelated	1 (5.6)	9 (27.3)	3 (17.6)	
Mismatched related	0 (0.0)	4 (12.1)	1 (5.9)	
Mismatched unrelated	2 (11.2)	2 (6.1)	1 (5.9)	
**Stem cell source**				0.496
Peripheral blood	14 (77.8)	18 (54.5)	11 (64.7)	
Bone marrow	1 (5.6)	6 (18.2)	1 (5.9)	
Bone marrow and peripheral blood	3 (16.7)	9 (27.3)	5 (29.4)	
**GVHD**				<0.001
Acute GVHD Grade 0-2	7 (38.9)	31 (93.9)	17 (100.0)	
Acute GVHD Grade 3-4 or Chronic GVHD	11 (61.1)	2 (6.1)	0 (0.0)	

a x^2^ test or Fisher exact test

b ANONA

### Epidemiology of CMV or RSV infection

**Figure 1:**
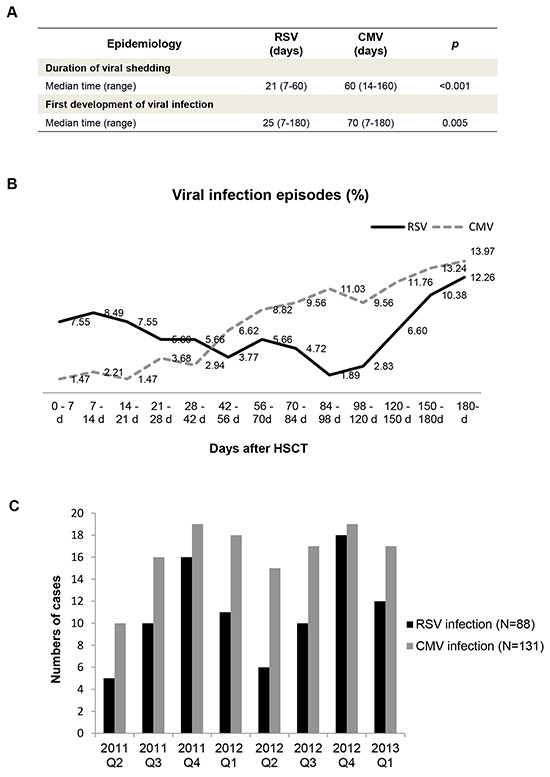
Epidemiology of RSV and CMV infection in hematopoietic stem cell transplant recipients **A.** Mann–Whitney *U* test was used to assess the statistical significance of the differences in the duration of viral shedding (21 VS 60, *p* < 0.001) and timing of the first development of viral infection (25 VS 70, *p* = 0.005) between CMV and RSV infections. **B.** The distribution of CMV (n = 131) and RSV (n = 88) infections according to number of days after HSCT. **C.** The distribution of CMV (n = 131) and RSV (n = 88) infections according to seasons from 2011 to 2013.

The median duration of viral shedding during RSV infections was 21 days (range: 7–60 days), while the median duration of CMV infection was 60 days (range: 14–160 days) (*p* < 0.001) (Figure 1A). In this cohort, RSV infection occurred earlier than CMV infection after HSCT (*p* = 0.005). Figure 1B shows that CMV infection rate steadily increased throughout the study period, while RSV infection rate was at a medium level at 0-28 days after HSCT, then decreased and occasionally increased for the remainder of the study period. The seasonal characteristics of CMV and RSV infection were further analysed. As shown in Figure 1C, the incidence of RSV infection was higher in winter and spring (*p* = 0.029). CMV infection rate was evenly distributed throughout the seasons of 2012.

### The correlation of CMV and RSV co-infection and severe pneumonia

This survey found that 16 (16/68; 23.5%) of the allogeneic HSCT patients were diagnosed with severe pneumonia. In the 18 allogeneic HSCT recipients with CMV and RSV co-infection, 11 patients (11/16; 68.8%) developed severe pneumonia during the study episode, whereas 5 cases (5/16; 31.2%) in the 33 patients with CMV/RSV mono-infection and no cases (0/16; 0.0%) in the 17 patients with no infection were diagnosed with severe pneumonia. The univariate analysis showed that age (≥28 years), gender (male), underlying diseases, and stem cell source were not associated with increased risk of severe pneumonia (Table 3). Acute GVHD grade 3-4 or chronic GVHD (OR 15.43; 95% CI 3.73-63.82; *p* < 0.001), bacterial infection (OR 5.46; 95% CI 1.26-23.70; *p* = 0.024) and CMV/RSV co-infection (OR 14.14; 95% CI 3.77-53.13; *p* < 0.001) were associated with the development of severe pneumonia in allogeneic HSCT patients. In a multivariable model, CMV and RSV co-infection (OR 5.75; 95% CI 1.07-30.97; *p* = 0.042) remained significant risk factor for progression to severe pneumonia (Table 4).

**Table 3:** Risk factors evaluated for the influence on severe pneumonia

Factor	Severe pneumonia	No severe pneumonia	OR (95% CI)	*P*
	(N=16)	(n=52)		
	N (%)	N (%)		
**Age** · **Years**				
<28	7 (43.8)	23 (44.2)	1.0	
≥28	9 (56.3)	29 (55.8)	1.02 (0.33-3.15)	0.973
**Gender**				
Female	4 (25.0)	21 (40.4)	1.0	
Male	12 (75.0)	31 (59.6)	2.03 (0.58-7.16)	0.270
**Underlying disease**				
Acute myelocytic leukemia	6 (37.5)	31 (59.6)	1.0	
Acute lymphoblastic leukemia	8 (50.0)	17 (32.7)	2.43 (0.72-8.17)	0.151
Chronic myelocytic leukemia	1 (6.3)	1 (1.9)	5.17 (0.28-94.50)	0.268
Others	1 (6.3)	3 (5.8)	1.72 (0.15-19.49)	0.661
**Donor type**				
Matched related	12 (75.0)	33 (63.5)	1.0	
Matched unrelated	2 (12.5)	11 (21.2)	0.83 (0.19-3.52)	0.795
Mismatched related	1 (6.3)	4 (7.7)	0.69 (0.07-6.78)	0.748
Mismatched unrelated	1 (6.3)	4 (7.7)	–	0.999
**Stem cell source**				
Bone marrow	1 (6.3)	7 (13.5)	1.0	
Peripheral blood	11 (68.8)	32 (61.5)	2.41 (0.27-21.81)	0.435
Bone marrow and peripheral blood	4 (25.0)	13 (25.0)	2.15 (0.20-23.18)	0.527
**GVHD**				
Acute GVHD Grade 0-2	7 (43.8)	48 (92.3)	1.0	
Acute GVHD Grade 3-4 or Chronic GVHD	9 (56.3)	4 (7.7)	15.43 (3.73-63.82)	<0.001
**Viral infection**				
CMV/RSV mono-infection or no infection	5 (31.3)	45 (86.5)	1.0	
CMV and RSV co-infection	11 (68.8)	7 (13.5)	14.14 (3.77-53.13)	<0.001
**Bacterial infection*^a^***				
No	11 (68.8)	48 (92.3)	1.0	
Yes	5 (31.3)	4 (7.7)	5.46 (1.26-23.70)	0.024

a Gram-negative bacillus and gram-positive coccus. Abbreviations: CMV, cytomegalovirus; RSV, respiratory syncytial virus; GVHD, graft vs host disease; OR, Odds ratio; CI, Confidence interval.

**Table 4:** Multivariable analysis of associated factors for severe pneumonia

Factor	OR (95% CI)	*P*
**GVHD**		
Acute GVHD Grade 0-2	1.0	
Acute GVHD Grade 3-4 or Chronic GVHD	4.95 (0.88-27.77)	0.069
**Viral infection**		
CMV/RSV mono-infection or no infection	1.0	
CMV and RSV co-infection	5.75 (1.07-30.97)	0.042
**Bacterial infection**		
No	1.0	
Yes	1.30 (0.19-8.97)	0.788

Abbreviations: CMV, cytomegalovirus; RSV, respiratory syncytial virus; GVHD, graft vs host disease; OR, Odds ratio; CI, Confidence interval.

### Mortality

**Figure 2:**
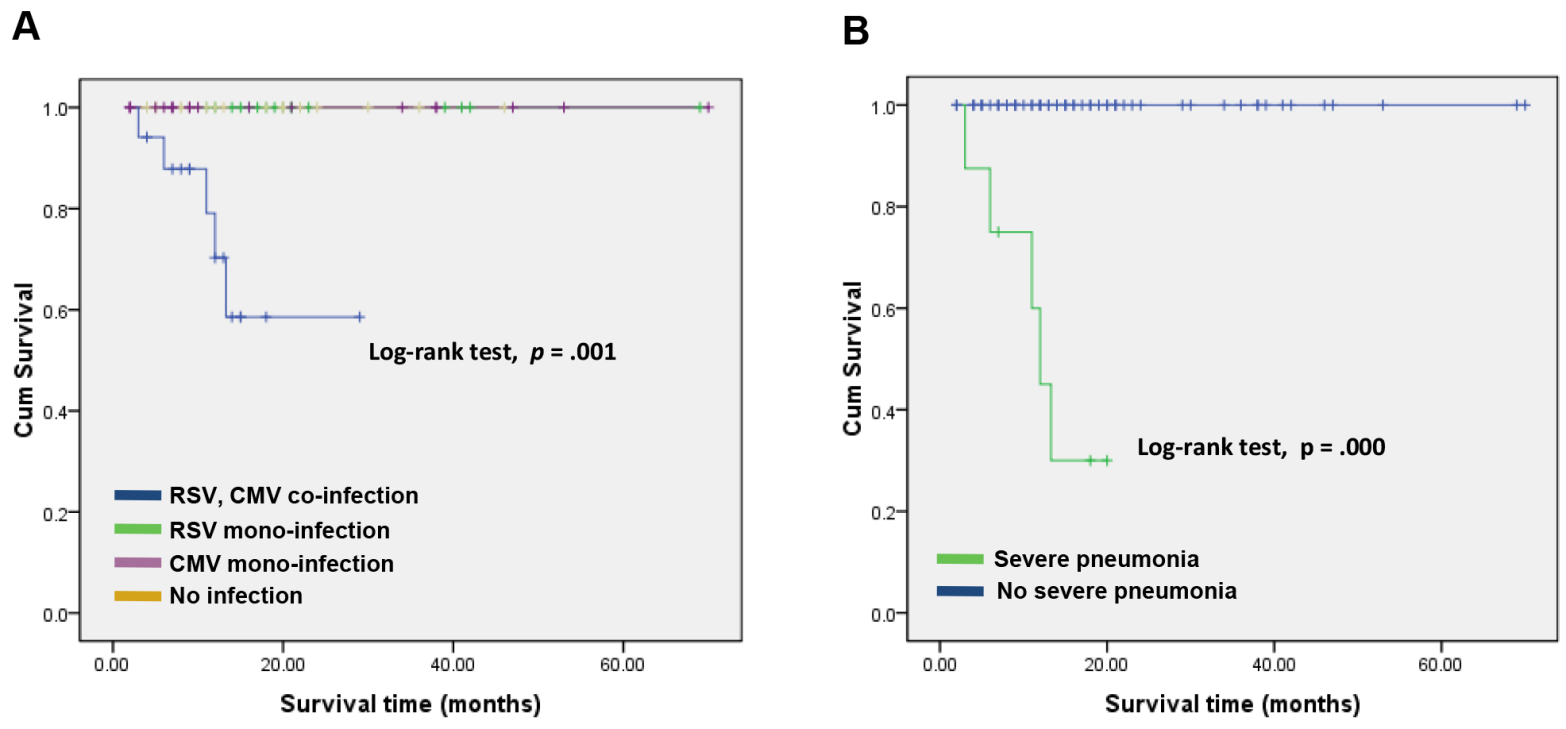
Kaplan-Meier estimation of overall survival in patients who developed CMV and RSV co-infection **A.** and severe pneumonia **B.** Co-infection of CMV and RSV (n=18) was closely correlated with poor overall survival compared to CMV or RSV mono-infection (n=33) and no infection (n=17) (*p* = 0.003) (A). The patients with severe pneumonia (n = 16) or not (n = 52) were strongly correlated with poor overall survival (*p* < 0.001) (B).

Five (5/68; 7.4%) HSCT patients died of severe pneumonia (median survival time was 206 days). The survival rates of allogeneic HSCT patients were 98.5% (67/68) at 100 days and 95.6% (65/68) at 1 year. The median survival time was 350 (90-1060) days in patients with CMV and RSV co-infection, 693 (110-1611) days in patients with mono-infections, and 696 (30-2033) days in patients with no infection. Figure 2 shows statistically significant differences in the overall survival rates of the three groups of patients (Log-rank trend, *p* = 0.001) (Figure 2A). Recipients who developed severe pneumonia (90-330; median 273 days) showed a significant survival disadvantage compared with patients who did not develop severe pneumonia (30-2033; median 511 days) (Log-rank trend, *p* < 0.001) (Figure 2B).

## DISCUSSION

Severe pneumonia leads more frequently to organ failure and death than other infections. In this study, the correlation of CMV/RSV co-infection and severe pneumonia was investigated in 68 allogeneic HSCT patients with hematologic malignancies. We observed a high incidence of severe pneumonia in the co-infection group (11/18) and a lower incidence in patients without co-infection (5/50). CMV and RSV co-infection was significantly associated with severe pneumonia and prognosis of allogeneic HSCT patients with hematologic malignancies.

To our knowledge, RSV and CMV are very different types of virus. RSV is a single-stranded RNA virus of the family Paramyxoviridae and well known to be seasonal [23]. It is a major cause of lower respiratory tract infections during infancy and childhood. CMV, a DNA virus of the Herpesviridae family [24], is one of the main agents involved in infectious complications after transplantation [25]. The occurrence of RSV infection was earlier than that of CMV, and RSV infections had a shorter duration of viral shedding after HSCT than CMV infection. It's well known that virus infection experiences three processes, including latent infection, active infection and disease [26]. The disease caused by viruses is due to transmission from the transplanted organ, reactivation of latent infection, or after a primary infection in seronegative transplant patients [27]. Simple RSV or CMV infection is mostly in the active period without signs and symptoms of the disease. However, co-infection will prolong defects in cellular immune function and accelerate the pathological process of infection with increasing the chemokine level and neutropenia [28, 29]. Moreover, the signaling pathways associated to innate and acquired immune system will be stimulated by co-infection of CMV and RSV. The innate cellular response to the initial stages of infection is mediated by Toll like receptor (TLR2, TLR4, TLR9) signaling, these receptors have been shown to recognize the surface proteins of CMV and RSV [30–32]. Additionally, both the RSV and CMV skewed CD4+ and CD8+ T cell activation and increased the inflammatory cytokines such as TNFα, IL-2, and IL6 etc.[28, 29, 33–36]. RSV NS1 protein partly suppresses human dendritic cell (DC) maturation [37] and CMV UL141 inhibits natural killer cell-mediated cytolysis by down-regulating cell surface expression of CD155 and CD112 [38, 39]. All above may be the reason that single virus infection does not significantly associated with severe pneumonia but co-infection does.

Prior studies have focussed on the importance of viral co-infection. Co-infection decreased the patient's response to antiviral treatment and resulted in more severe infection and a greater risk of death. CMV/BKV co-infection (7/8; 87.5%) is the major cause of death among patients with viral co-infection [40]. Furthermore, co-infection with CMV/EBV is associated with poor survival and a greater risk of post-transplantation complications [41]. Virus co-infection induced bronchial epithelial damage, loss of clearance, and pathogen invasion.

Recent advances in diagnostic techniques such as CMV antigenaemia quantification and PCR enable the early detection of CMV reactivation and prevent the subsequent development of CMV diseases [42]. Unfortunately, there is no strategy for addressing RSV associated complications. HSCT patients, especially those with co-infection, should be closely monitored to prevent the development of severe pneumonia. Prophylactic Acyclovir could not prevent co-infection with CMV and RSV because of the dose of Acyclovir, patient sensitivity, and viral variation. Further study is required to establish effective anti-viral prophylactic treatment after allogeneic HSCT.

Pneumonia is caused by more than one pathogens (e.g. bacterial and viral) and co-existence in HSCT patients. Bacterial infections continue to be one of the most frequent complications after HSCT [43]. We also adopted for the investigation of bacterial infection in the patients. In univariate analysis, we found that bacterial infection was associated with severe pneumonia, but was not significant risk factor in multivariable analysis.

This study had several potential limitations. First, the number of patients was small because it was difficult to enrol large number of patients from a single transplant centre. Second, T-cell depletion, antilymphocyte globulin, unrelated or HLA mismatched donors, and GVHD are known to be risk factors for severe viral infections after HSCT [26, 44, 45]. In this study, T-cell depletion was not assessed and antilymphocyte globulin assays were not performed. Finally, PCR assays were performed on oropharynx swabs not bronchoalveolar lavage fluid. This limited the frequency with which the quantitative PCR tests could be performed. Nonetheless, these data provided new information about the risk factor of CMV/RSV co-infection and the correlation between CMV/RSV co-infection and severe pneumonia in allogeneic HSCT recipients.

In conclusion, this study found that co-infection with CMV and RSV facilitated the development of severe pneumonia in allogeneic HSCT patients with underlying hematologic malignancies. These results indicate that further investigation into viral co-infection following HSCT is necessary and helpful to predict the incidence of severe pneumonia and prognosis.

## MATERIALS AND METHODS

### Patients

A total of 437 oropharynx swabs and 437 peripheral blood samples were collected from 68 recipients who underwent allogeneic HSCT at the first affiliated hospital, Sun Yat-sen University, Guangzhou, China, from March 2011 to February 2013 (preliminary data for patients are shown in Table 1 ). Samples were tested regardless of the presence of symptoms of respiratory infection. Most of allogeneic HSCT patients were monitored every two weeks for six months (once a week in the first month). The CMV serology status of the donor was tested before HSCT. The donors were all positive for CMV-IgG and negative for CMV-IgM. Clinical and laboratory data were abstracted from the existing electronic medical records. Bacterial infections were detected in blood, tracheal secretions or broncho-alveolar lavage.

### CMV-IgG and CMV-IgM detection

The assays were performed using the Abbott Laboratories Microparticle enzyme immunoassay on an Architech C16000. Briefly, the microparticle bottle was mixed to suspend the microparticles. The minimum sample cup volume was calculated and detected. The detection of CMV IgG indicated a previous infection with CMV, and anti-CMV IgM ≥1.0 reflected either an active recent infection or reactivation of the virus.

### Quantification of CMV DNA copy number

The detection of CMV DNA was performed using the Diagnostic Kit for Quantification of Human Cytomegalovirus DNA (Daangene, Guangzhou, China). Peripheral blood leukocyte DNA was extracted and added to the CMV-PCR reaction tube. The negative control and positive quantification controls were installed. Quantitative-PCR was performed in an ABI Prism 7000 machine using the following protocol: 2 min at 93°C, 10 cycles of 45 s at 93°C, 60 s at 55°C, and 30 cycles of 30 s at 93°C and 45 s at 55°C.

### Quantitative PCR analysis of RSV

Ribonucleic acids were isolated from 1 mL of the oropharynx specimens. A nucleic acid extraction method (QIAamp Viral RNA Mini kit; Qiagen, Hilden, Germany) was used. RSV primers: 5′- TGA ACA ACC CGC ATC ATT- 3′ (911~932 bp), 5′- GCA TTG CCT AAT ACT ACA CTA GAG AA- 3′ (983~958 bp); Taqman Probe N: 5′- FAM-CTT TGA CTC AAT TTC CC-MGB- 3′ RT-PCR was performed in an Applied Biosystems 7500 machine using the following protocol: 2 min at 95°C, 15 s at 94°C, 15 s at 55°C, and 20 s at 72°C, followed by 50 cycles of 20 sec at 94°C and 35 sec at 55°C. If the cumulative curve was S-shaped and the CT value was <30, the sample was determined to be positive [21].

### Virus prophylaxis

Ganciclovir (5 mg/kg i.v. q12h daily) was given prophylactically to all HSCT patients from the day that conditioning chemotherapy was initiated until day 1 after allogeneic HSCT, followed by Acyclovir (200 mg P.O. 3 times daily) for 6 months. If the CMV viral load exceeded 500 copies/mL or the CMV IgM was ≥1.0, indicating CMV reactivation, Ganciclovir (5 mg/kg i.v. q12h daily) was used instead of Acyclovir until the patient's parameters improved (CMV viral load <500 copies/mL or CMV IgM <1.0). Allogeneic HSCT patients received intravenous immunoglobulin (0.2 g/kg every month) for 6 months.

### Criteria for analysis and definitions

Mono-infection was defined by the detection of CMV reactivation or detection of RSV in the period lasting more than two weeks. Co-infection was defined by the detction of both CMV reactivation and RSV infection in two weeks. The definitions for severe pneumonia (severe community-acquired pneumonia) included five “minor” criteria and four “major” criteria. The minor criteria included respiratory rate >30/min, bilateral pneumonia or multilobar pneumonia, systolic BP <90 mm Hg, and diastolic BP <60 mm Hg. The major criteria included a need for mechanical ventilation, an increase in the size of infiltrates by ≥50% within 48 h, either septic shock or the need for pressors for >4 h, and acute renal failure (either urine output <80 ml in 4 h or serum creatinine levels >2 mg/dl in the absence of chronic renal failure) [46, 47].

### Statistical analysis

To assess associations between continuous variables and the outcome variable, Mann–Whitney *U* test or ANOVA was used. The Chi-square test or Fisher's exact test was used to analyse categorical data, as appropriate. Logistic regression models were used to estimate the associations of potential risk factors for severe pneumonia. Kaplan-Meier was used for survival analysis, and the differences in survival probabilities between patient subsets were assessed by the log-rank test. Two-sided *p* values of less than 0.05 were considered to be statistically significant. Statistical analyses were performed using SPSS.20.0 software (SPSS, Inc, Chicago, IL).

## Abbreviations

HSCT: hematopoietic stem cell transplant
CMV: cytomegalovirus
RSV: respiratory syncytial virus
allogeneic HSCT: allogeneic hematopoietic stem cell transplant
GVHD: graft-versus-host disease
PIV: parainfluenza virus
MPV: metapneumovirus.

## References

[1] Copelan EA (2006). Hematopoietic stem-cell transplantation. N Engl J Med.

[2] Aguilar-Guisado M, Jimenez-Jambrina M, Espigado I, Rovira M, Martino R, Oriol A, Borrell N, Ruiz I, Martin-Davila P, de la Camara R, Salavert M, de la Torre J, Cisneros JM, Spanish Network for Research in Infectious D (2011). Pneumonia in allogeneic stem cell transplantation recipients: a multicenter prospective study. Clin Transplant.

[3] Roychowdhury M, Pambuccian SE, Aslan DL, Jessurun J, Rose AG, Manivel JC, Gulbahce HE (2005). Pulmonary complications after bone marrow transplantation: an autopsy study from a large transplantation center. Arch Pathol Lab Med.

[4] Lucena CM, Torres A, Rovira M, Marcos MA, de la Bellacasa JP, Sanchez M, Domingo R, Gabarrus A, Mensa J, Agusti C (2014). Pulmonary complications in hematopoietic SCT: a prospective study. Bone Marrow Transplant.

[5] Ruuskanen O, Lahti E, Jennings LC, Murdoch DR (2011). Viral pneumonia. Lancet.

[6] Chen J, Hu L, Wu M, Zhong T, Zhou YH, Hu Y (2012). Kinetics of IgG antibody to cytomegalovirus (CMV) after birth and seroprevalence of anti-CMV IgG in Chinese children. Virol J.

[7] George B, Pati N, Gilroy N, Ratnamohan M, Huang G, Kerridge I, Hertzberg M, Gottlieb D, Bradstock K (2010). Pre-transplant cytomegalovirus (CMV) serostatus remains the most important determinant of CMV reactivation after allogeneic hematopoietic stem cell transplantation in the era of surveillance and preemptive therapy. Transpl Infect Dis.

[8] Stocchi R, Ward KN, Fanin R, Baccarani M, Apperley JF (1999). Management of human cytomegalovirus infection and disease after allogeneic bone marrow transplantation. Haematologica.

[9] Boeckh M, Ljungman P (2009). How we treat cytomegalovirus in hematopoietic cell transplant recipients. Blood.

[10] Peck AJ, Englund JA, Kuypers J, Guthrie KA, Corey L, Morrow R, Hackman RC, Cent A, Boeckh M (2007). Respiratory virus infection among hematopoietic cell transplant recipients: evidence for asymptomatic parainfluenza virus infection. Blood.

[11] Harrington RD, Hooton TM, Hackman RC, Storch GA, Osborne B, Gleaves CA, Benson A, Meyers JD (1992). An outbreak of respiratory syncytial virus in a bone marrow transplant center. J Infect Dis.

[12] Martinez FD (2003). Respiratory syncytial virus bronchiolitis and the pathogenesis of childhood asthma. Pediatr Infect Dis J.

[13] Murata Y (2008). Respiratory syncytial virus infection in adults. Curr Opin Pulm Med.

[14] Nair H, Nokes DJ, Gessner BD, Dherani M, Madhi SA, Singleton RJ, O'Brien KL, Roca A, Wright PF, Bruce N, Chandran A, Theodoratou E, Sutanto A (2010). Global burden of acute lower respiratory infections due to respiratory syncytial virus in young children: a systematic review and meta-analysis. Lancet.

[15] Whimbey E, Champlin RE, Englund JA, Mirza NQ, Piedra PA, Goodrich JM, Przepiorka D, Luna MA, Morice RC, Neumann JL (1995). Combination therapy with aerosolized ribavirin and intravenous immunoglobulin for respiratory syncytial virus disease in adult bone marrow transplant recipients. Bone Marrow Transplant.

[16] Erard V, Storer B, Corey L, Nollkamper J, Huang ML, Limaye A, Boeckh M (2004). BK virus infection in hematopoietic stem cell transplant recipients: frequency, risk factors, and association with postengraftment hemorrhagic cystitis. Clin Infect Dis.

[17] Weigt SS, Gregson AL, Deng JC, Lynch JP, Belperio JA (2011). Respiratory viral infections in hematopoietic stem cell and solid organ transplant recipients. Semin Respir Crit Care Med.

[18] Ustun C, Slaby J, Shanley RM, Vydra J, Smith AR, Wagner JE, Weisdorf DJ, Young JA (2012). Human parainfluenza virus infection after hematopoietic stem cell transplantation: risk factors, management, mortality, and changes over time. Biol Blood Marrow Transplant.

[19] Buyck HC, Prentice HG, Griffiths PD, Emery VC (2010). The risk of early and late CMV DNAemia associated with Campath use in stem cell transplant recipients. Bone Marrow Transplant.

[20] Ghosh S, Champlin RE, Englund J, Giralt SA, Rolston K, Raad I, Jacobson K, Neumann J, Ippoliti C, Mallik S, Whimbey E (2000). Respiratory syncytial virus upper respiratory tract illnesses in adult blood and marrow transplant recipients: combination therapy with aerosolized ribavirin and intravenous immunoglobulin. Bone Marrow Transplant.

[21] Raboni SM, Nogueira MB, Tsuchiya LR, Takahashi GA, Pereira LA, Pasquini R, Siqueira MM (2003). Respiratory tract viral infections in bone marrow transplant patients. Transplantation.

[22] Avetisyan G, Mattsson J, Sparrelid E, Ljungman P (2009). Respiratory syncytial virus infection in recipients of allogeneic stem-cell transplantation: a retrospective study of the incidence, clinical features, and outcome. Transplantation.

[23] Bennett N, Ellis J, Bonville C, Rosenberg H, Domachowske J (2007). Immunization strategies for the prevention of pneumovirus infections. Expert Rev Vaccines.

[24] Varani S, Landini MP (2011). Cytomegalovirus-induced immunopathology and its clinical consequences. Herpesviridae.

[25] Azevedo LS, Pierrotti LC, Abdala E, Costa SF, Strabelli TM, Campos SV, Ramos JF, Latif AZ, Litvinov N, Maluf NZ, Caiaffa Filho HH, Pannuti CS, Lopes MH (2015). Cytomegalovirus infection in transplant recipients. Clinics (Sao Paulo).

[26] Ljungman P, Griffiths P, Paya C (2002). Definitions of cytomegalovirus infection and disease in transplant recipients. Clin Infect Dis.

[27] Gandhi MK, Khanna R (2004). Human cytomegalovirus: clinical aspects, immune regulation, and emerging treatments. Lancet Infect Dis.

[28] Chen YC, Chiang HH, Cho YT, Chang CY, Chen KL, Yang CW, Lee YH, Chu CY (2015). Human herpes virus reactivations and dynamic cytokine profiles in patients with cutaneous adverse drug reactions –a prospective comparative study. Allergy.

[29] Fishman JA (2011). Infections in immunocompromised hosts and organ transplant recipients: essentials. Liver Transpl.

[30] Boehme KW, Guerrero M, Compton T (2006). Human cytomegalovirus envelope glycoproteins B and H are necessary for TLR2 activation in permissive cells. J Immunol.

[31] Compton T, Kurt-Jones EA, Boehme KW, Belko J, Latz E, Golenbock DT, Finberg RW (2003). Human cytomegalovirus activates inflammatory cytokine responses via CD14 and Toll-like receptor 2. J Virol.

[32] Polack FP, Irusta PM, Hoffman SJ, Schiatti MP, Melendi GA, Delgado MF, Laham FR, Thumar B, Hendry RM, Melero JA, Karron RA, Collins PL, Kleeberger SR (2005). The cysteine-rich region of respiratory syncytial virus attachment protein inhibits innate immunity elicited by the virus and endotoxin. Proc Natl Acad Sci U S A.

[33] Bitmansour AD, Douek DC, Maino VC, Picker LJ (2002). Direct ex vivo analysis of human CD4(+) memory T cell activation requirements at the single clonotype level. J Immunol.

[34] Munir S, Hillyer P, Le Nouen C, Buchholz UJ, Rabin RL, Collins PL, Bukreyev A (2011). Respiratory syncytial virus interferon antagonist NS1 protein suppresses and skews the human T lymphocyte response. PLoS Pathog.

[35] Gamadia LE, Rentenaar RJ, van Lier RA, ten Berge IJ (2004). Properties of CD4(+) T cells in human cytomegalovirus infection. Hum Immunol.

[36] Isaacson MK, Juckem LK, Compton T (2008). Virus entry and innate immune activation. Curr Top Microbiol Immunol.

[37] Munir S, Le Nouen C, Luongo C, Buchholz UJ, Collins PL, Bukreyev A (2008). Nonstructural proteins 1 and 2 of respiratory syncytial virus suppress maturation of human dendritic cells. J Virol.

[38] Prod'homme V, Griffin C, Aicheler RJ, Wang EC, McSharry BP, Rickards CR, Stanton RJ, Borysiewicz LK, Lopez-Botet M, Wilkinson GW, Tomasec P (2007). The human cytomegalovirus MHC class I homolog UL18 inhibits LIR-1+ but activates LIR-1- NK cells. J Immunol.

[39] Prod'homme V, Sugrue DM, Stanton RJ, Nomoto A, Davies J, Rickards CR, Cochrane D, Moore M, Wilkinson GW, Tomasec P (2010). Human cytomegalovirus UL141 promotes efficient downregulation of the natural killer cell activating ligand CD112. J Gen Virol.

[40] Watcharananan SP, Kiertiburanakul S, Piyatuctsanawong W, Anurathapan U, Sungkanuparph S, Pakakasama S, Chantratita W, Hongeng S (2010). Cytomegalovirus, adenovirus, and polyomavirus co-infection among pediatric recipients of allogeneic stem cell transplantation: characteristics and outcome. Pediatr Transplant.

[41] Jaskula E, Dlubek D, Sedzimirska M, Duda D, Tarnowska A, Lange A (2010). Reactivations of cytomegalovirus, human herpes virus 6, and Epstein-Barr virus differ with respect to risk factors and clinical outcome after hematopoietic stem cell transplantation. Transplant Proc.

[42] Mori T, Kato J (2010). Cytomegalovirus infection/disease after hematopoietic stem cell transplantation. Int J Hematol.

[43] Balletto E, Mikulska M (2015). Bacterial Infections in Hematopoietic Stem Cell Transplant Recipients. Mediterranean journal of hematology and infectious diseases.

[44] Nachbaur D, Larcher C, Kircher B, Eibl G, Nussbaumer W, Gunsilius E, Haun M, Grunewald K, Gastl G (2003). Risk for cytomegalovirus infection following reduced intensity allogeneic stem cell transplantation. Ann Hematol.

[45] van Burik JA, Carter SL, Freifeld AG, High KP, Godder KT, Papanicolaou GA, Mendizabal AM, Wagner JE, Yanovich S, Kernan NA (2007). Higher risk of cytomegalovirus and aspergillus infections in recipients of T cell-depleted unrelated bone marrow: analysis of infectious complications in patients treated with T cell depletion versus immunosuppressive therapy to prevent graft-versus-host disease. Biol Blood Marrow Transplant.

[46] Mandell LA (2009). Severe community-acquired pneumonia (CAP) and the Infectious Diseases Society of America/American Thoracic Society CAP guidelines prediction rule: validated or not. Clin Infect Dis.

[47] Mandell LA, Wunderink RG, Anzueto A, Bartlett JG, Campbell GD, Dean NC, Dowell SF, File TM, Musher DM, Niederman MS, Torres A, Whitney CG, Thoracic S, Infectious Diseases Society of A and American (2007). Infectious Diseases Society of America/American Thoracic Society consensus guidelines on the management of community-acquired pneumonia in adults. Clin Infect Dis.

